# Online scheduling using a fixed template: the case of outpatient chemotherapy drug administration

**DOI:** 10.1007/s10729-022-09616-1

**Published:** 2022-11-02

**Authors:** Alireza F. Hesaraki, Nico P. Dellaert, Ton de Kok

**Affiliations:** 1 Queen’s Management School, Queen’s University Belfast, Belfast, BT9 5EE UK; 2 School of Industrial Engineering, Eindhoven University of Technology, PO Box 513, 5600 MB Eindhoven, The Netherlands; 3 Centrum Wiskunde & Informatica (CWI), 1098 XG Amsterdam, The Netherlands

**Keywords:** Online scheduling, Template, Mixed integer programming, Simulation, Chemotherapy, Operations research, Operations management

## Abstract

In this paper, we use a fixed template of slots for the online scheduling of appointments. The template is a link between planning the service capacity at a tactical level and online scheduling at an operational level. We develop a detailed heuristic for the case of drug administration appointments in outpatient chemotherapy. However, the approach can be applied to online scheduling in other application areas as well. The desired scheduling principles are incorporated into the cost coefficients of the objective function of a binary integer program for booking appointments in the template, as requests arrive. The day and time of appointments are decided simultaneously, rather than sequentially, where optimal solutions may be eliminated from the search. The service that we consider in this paper is an example to show the versatility of a fixed template online scheduling model. It requires two types of resource, one of which is exclusively assigned for the whole appointment duration, and the other is shared among multiple appointments after setting up the service. There is high heterogeneity among appointments on a day of this service. The appointments may range from fifteen minutes to more than eight hours. A fixed template gives a pattern for the scheduling of possibly required steps before the service. Instead of maximizing the fill-rate of the template, the objective of our heuristic is to have high performance in multiple indicators pertaining to various stakeholders (patients, nurses, and the clinic). By simulation, we illustrate the performance of the fixed template model for the key indicators.

## Introduction

The goal for appointment scheduling in many service industries is to efficiently use resources and avoid queuing [27]. Online scheduling and offline scheduling are two main approaches for responding to appointment requests. When making decisions in *online scheduling*, only one or some of the appointment requests for the day to be scheduled are known. The appointment day and time can be determined either immediately upon request or shortly afterwards, e.g., within 24 hours. In *offline scheduling*, all requests for the day to be scheduled are known at the decision moment. Hence, to generate a high performing schedule, the time of appointment is not determined as early as in online scheduling, i.e., not until a couple of days before the intended appointment day. Nevertheless, the appointment day may immediately be determined.

In online scheduling, the job data are not known until the job is *released*, i.e., the appointment request is received by the service provider. This lack of information a priori makes it challenging to optimize online schedules. In this paper, we consider a *clairvoyant online-over-time* problem, where jobs are going to be released at different points in time, but when a job is released all its data are known [26].

If appointments were made using the first-come-first-served (FCFS) discipline, the resulting schedule would depend on the order in which the requests arrive: job permutation. Each appointment would be placed to start at the earliest opportunity when the required resources are available for the job, e.g., a nurse and a chair or bed for drug administration. The FCFS discipline can result in a long makespan (overtime) and low resource utilization. FCFS also cannot incorporate the time requirements or interests of stakeholders, e.g., oncologists, pharmacy, patients, and the clinic.

Appointment templates are arrangements of vacant appointment slots optimized for some criteria. It is common practice to use templates in online scheduling [22]. In contrast to FCFS, a template helps to efficiently use resources while incorporating the interests of stakeholders by systematically managing the starting time of each appointment. In particular, with a template, resources are allocated at an operational level based on various appointment durations and their estimated demands. Thus, the duration of slots and their frequencies in a template are determined based on information about demand, e.g., relative frequency of appointment durations of the past year and forecasted annual number of appointments. The time and resource arrangement of vacant slots in the template is optimized for some criteria, such as makespan and flowtime.

For healthcare services, well-designed appointment systems specify various access rules that determine when which types of patients may access the available care providers. In specialty care services such as chemotherapy, the preferences of both patients and care providers have to be taken into consideration [9]. The patients’ waiting time is an important criterion for improving the quality of outpatient services. Having short waiting times is a competitive advantage among service providers when on the one hand patients’ expectations have increased through searching information, and on the other hand, the trend toward more outpatient services can increase waiting times. Policies have also often neglected waiting times in favor of care providers [17]. The idle time and overtime of care providers have to be balanced against the waiting time of patients [19, 29].

Chemotherapy is a common procedure against cancer, next to radiotherapy and surgery. For every patient being treated with chemotherapy, a treatment protocol (aka regimen) is prescribed. The protocol determines the chemotherapeutic agents (drugs), doses, delivery method (topical, ingestion, injection, infusion), duration of infusion, planned days of drug administration in cyclical periods, and the number and duration of cycles that are repeated back-to-back during the treatment.

In the drug administration of outpatient chemotherapy, both a nurse and a chair or bed are required at every moment of the service. There are also hard constraints on the tasks of nurses. These requirements make the service an interesting problem to study the performance of online scheduling using a fixed template. For brief reviews of the literature on scheduling outpatient chemotherapy appointments, we refer the reader to [18, 21], and [33].

More recently, Benzaid et al. [2] develop a two-stage mathematical formulation. In the first stage, at the end of each day, they schedule the patient appointments and estimate the number of required nurses for that schedule. The objective is to maximize the total number of scheduled appointments. In the second stage, they assign patients to the minimum number of nurses required. Slocum et al. [32] present a case study where discrete-event simulation shows a reduction in average waiting time and average overtime due to dividing the patients into two or three categories based on the appointment durations. Alvarado and Ntaimo [1] use stochastic programming for scheduling all the appointments of a patient’s treatment, and [3] use stochastic programming to fine-tune the appointment times of an online schedule, a day or two before the schedule is implemented. However, in both studies, the models are applied to small instances: about 24 and 12 patients per day. Demir et al. [5] formulate a stochastic program to minimize the expected weighted sum of overtime, idle time, and waiting time, but they also solve their model for instances with few patients. Mandelbaum et al. [23] reduce waiting and overtime in the offline scheduling problem for a large clinic with roughly 90 appointments per day by a heuristic based on an infinite-server queuing model.

Hahn-Goldberg et al. [10] and Condotta and Shakhlevich [4] consider online appointment scheduling for chemotherapy using random appointment templates that are generated using historical information about appointments at the clinics. Hahn-Goldberg et al. [10] generate a new template around the booked appointments when an appointment request does not fit into any of the vacant slots of the current template, whereas Condotta and Shakhlevich [4] generate a new template each time a request arrives.

Hahn-Goldberg et al. [10] do not give any procedure for deciding the day of appointments. They assume that when an appointment request arrives, its day has already been decided and the time of the appointment must be booked online for that day. They use makespan minimization as the objective for generating the random templates.

Condotta and Shakhlevich [4], determine the day, time, and nurse for each request that arrives. They solve the day and time problems consecutively, and assign the same nurse to the patient for the whole appointment. When there is a “clash” between two patients assigned to the same nurse, they resolve it with a special arrangement by an additional nurse.

**Fig. 1:**
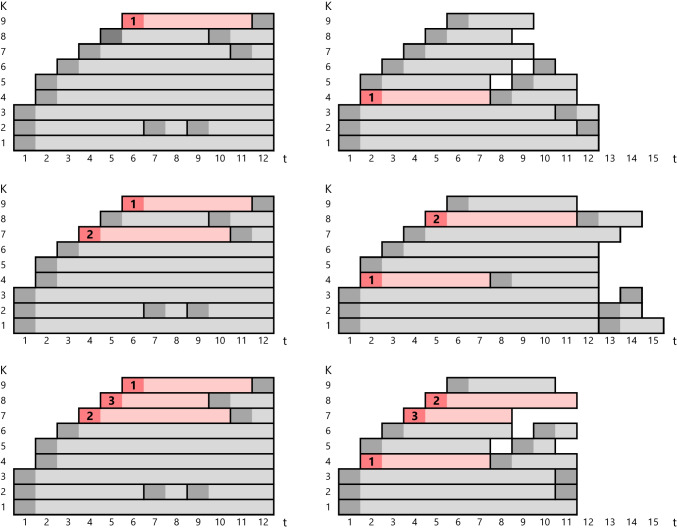
Fixed template (left) versus random templates (right): randomly drawn slots are arranged around the already booked appointments while the total number of slots is the same as the fixed template: *K* = 9 stations and *P* = 14 slots, and *T* relaxed rather than fixed to 12 timeslots

In contrast to random templates, a *fixed template* gives a *pattern* for the scheduling of short preparatory steps prior to the service, e.g., drug preparation, while incorporating a few time requirements in the design of the template. Hence, using a fixed template is effective and less confusing for the service provider. The difference between a fixed template and random templates is illustrated in Fig. 1, where one appointment is booked at a time. A new random template is generated each time a booking request arrives. First, a request for an appointment with duration *l* = 6 timeslots arrives and is scheduled, followed by two appointment requests with *l* = 7 and *l* = 5, respectively. In the fixed template on the left side of the figure, the slots are fixed: the same template is used and filled with new bookings in the current vacant slots. But in random templates on the right side, slots are randomly drawn from a multinomial distribution and then, using an optimization model or algorithm, arranged in the empty time-station space available around the already booked appointments. If none of the slots in the random template exactly fit the length of the appointment being booked, random template generation is repeated until there is a fit. The fixed template has *P* = 14 slots in total (either filled or vacant). For the random template, as many random slots are drawn to make the total (including the already booked slots) equal to *P* = 14.

**Fig. 2:**
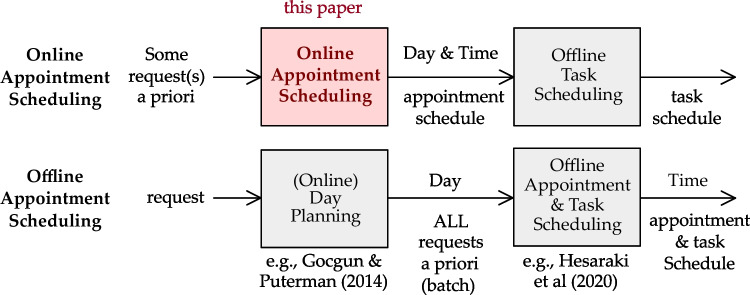
Flow of information in online and offline appointment scheduling (adopted from [13])

We propose a multi-criterion heuristic to systematically follow specified scheduling principles in using a fixed template of appointment slots to decide the day and time of appointments. In the stylized example settings that we will use in Section 5 for numerical illustration, because of patient heterogeneity and not having separate servers among the types of requests, queuing analysis of the problem is complicated. Hence, we use simulation to evaluate the steady-state performance of our heuristic. As we simultaneously determine the day and time of appointments in a single formulation rather than sequentially, a better schedule may be generated since it does not get eliminated by fixing the day before deciding the time of appointments. We do not allow patient overlaps (clashes) in the first place, because the template is designed based on hard constraints on the setting up and monitoring tasks of the nurses. Figure 2 shows the position of our model in the context of appointment scheduling. The service provider can assign the tasks offline because it is an internal matter in contrast to the appointment time, which involves the customer. After the online appointment schedule is finalized, e.g., a day before the appointments, the offline task schedule can be generated. Maximizing the fill-rate (fraction of template slots that are booked) is a common objective in the literature on online scheduling [22]. However, our objective is to have good performance for key indicators relevant to the service: little indirect waiting time^1^ for new patients, the appointments of returning patients scheduled as many as possible within their flexibility windows, and little overtime and idle time for the clinic.

## Problem definition

Chemotherapeutic drugs kill rapidly dividing cells. Most cancerous cells divide rapidly. However, some types of normal cells (e.g., bone marrow, the lining of the mouth, the gastrointestinal tract, and hair follicles) also divide rapidly, even in adults. Thus, chemotherapy comes with side effects on the normal cells as well, and it is therefore administered in multiple installments with carefully chosen doses. Moreover, if drugs are administered too early they will still have side effects on normal cells, and if they are administered too late, there will be diminishing results of the prior installment and consequently a regrowth of cancerous cells.

**Fig. 3:**
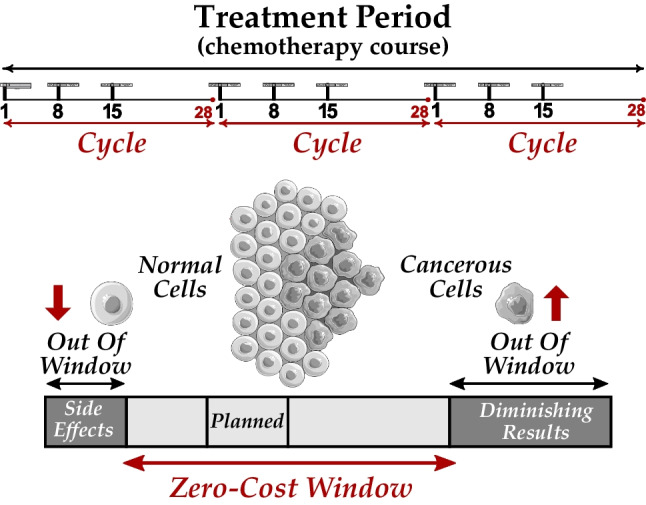
Above: a treatment period that includes three 28-day cycles and three planned days (1, 8, and 15) in each cycle. Below: a ZCW around a planned day extending from one day before to two days after

The patient’s treatment protocol indicates the number of days planned between turns of drug administration. However, the time period between appointments may have some flexibility as the goal is to strike a balance between effectiveness and side effects [8, 30]. For example, the next appointment can be equally held one day earlier or two days later than the planned day indicated in the cycle. Thus, there is a flexibility window around each planned day for drug administration, where any day is acceptable for scheduling the patient’s appointment at the clinic. Before the window, there are overdose side effects, and after the window, there are diminishing results of the previous drug administration installment. As shown in Fig. 3, we refer to the acceptable days as the zero-cost window (ZCW), since no cost (penalty) is enforced for those days in our scheduling model. The days that are out-of-window (OOW) should be avoided if possible: the fewer the violation, the better. As [7] points out, this is clinically critical: *“when chemotherapy is being given with curative intent, we believe that it is important to avoid reductions and delays in chemotherapy if the best possible outcome is to be achieved, although this is not possible for all patients.”*

Depending on the prescribed regimen, the effectiveness and side effects of the patient’s chemotherapy are checked via laboratory tests before some installments of drug administration, if not before each of them. If the test results are unsatisfactory, the treating oncologist or a nurse decides to postpone the appointment (e.g., for a week), or the treatment may continue with another regimen. Otherwise, an order is sent to the pharmacy to prepare the drugs, and subsequently nurses administer the drugs. Some clinics hold the laboratory test and oncologist visit one or two days before drug administration and others on the same day. Depending on the price or shelf-life, the drugs of some regimens may already be prepared before the day of drug administration [24, 36]. However, for many regimens, the drugs must be prepared on the same day.

The staffing cost is the second largest cost, after drugs, at oncology clinics [21]. Also, the nursing capacity is a critical bottleneck of patient flow in oncology treatments, including outpatient chemotherapy [37]. Time pressure and the lack of trained nurses are the main causes of chemotherapy administration errors [25, 35]. To avoid the risks associated with multitasking, hard constraints are enforced on the nursing tasks (setting up and monitoring), which further limit utilization of the nursing capacity. Hence, it is important to carefully schedule appointments for high-utilization of the available nursing capacity. In online scheduling, this can be achieved by using an appointment template based on the available number of nurses and infusion stations (chairs or beds and infusion pumps). For appointments where there is time limitation due to a preparatory step, priority slots can be designated in the template to satisfy the time requirements. An example of this kind of limitation is when certain drugs cannot be prepared in the morning or the treating oncologist must authorize infusion on the same day rather than the day before and is only available in the morning.

In the online scheduling of drug administration appointments, our goal is to have little indirect waiting time for new patients (time between referral to the clinic and their first appointment), as many appointments as possible scheduled in their ZCWs for returning patients, little overtime for nurses, and as few idle station-timeslots as possible in the template for the clinic. To this end, we develop a binary integer program (BIP) for placing appointments in a fixed template, which can be applied in two different modes of responding to requests: *immediately* and *daily*. In scheduling immediately, the appointment day and time is given to the patient right away, whenever a request arrives. In the daily mode, the day and time of the appointments are given to the patients at the end of the day on which the requests are made. Immediately scheduling is in favor of the patients as they know the appointment time as soon as they request it. In contrast, daily scheduling gives more flexibility to the clinic: further information can be utilized before the times are announced to the patients to possibly make last-minute adjustments to constraints and to have the scheduling requirements met as much as possible. We must note that the daily mode is also online scheduling because not all the patients of the days for which decisions are being made are known a priori. In this mode, the clinic waits until the end of the current day to collect all the requests that arrived on the current day—not all the requests for the days for which decisions are being made.

Scheduling immediately is associated with the unit arrival process outlined by [9]: the requests arrive one at a time and at random time lapses between them. We assume that the time lapses are exponentially distributed, i.e., a Poisson arrival process. The daily mode of scheduling is associated with the periodic arrival process that they describe. The appointment requests are accumulated over discrete time periods of 24 hours, and decisions are made at the end of the time periods. The inter-arrival times are constant, but the number and types of requests are random.

The time required for drug administration in chemotherapy ranges from fifteen minutes to more than eight hours among patients scheduled on a day in a clinic [34]. Various treatment protocols that have the same infusion duration can be grouped together as the same type that requires a certain appointment duration. This is considered at the tactical level when designing the appointment template. The relative frequency of appointments in the past year and the projected number of requests in the current year can be used to determine the number of slots needed with each duration in the template. Hesaraki et al. [12] consider templates with minimum flowtime and makespan as criteria to have appointments started and completed as early as possible for the sake of patients and nurses.

Besides appointment durations, two other features of the protocols are operationally relevant as they are used for appointment scheduling: the planned days of drug administration and the flexibility windows (ZCWs) around them. Each *request* from patient *p* is mainly characterized by the *desired day* ($d_{p}^{\prime }$) for the next appointment being requested, lengths of the two sides of the *ZCW* (*δ*_1,*p*_, *δ*_2,*p*_), and the *appointment duration* ($l_{p}^{\prime }$). We make a distinction between the desired day that is based on the days planned in the patient’s treatment protocol and the day that is actually scheduled for drug administration. The scheduled day may differ from the planned day, which is acceptable (no penalty in the BIP) as long as it is within its ZCW.

The opening hours of the clinic are divided into equal-duration unit-timeslots. The duration of every appointment is an integer number of unit-timeslots. In the remainder of the paper, we refer to these unit-timeslots simply as *timeslots*, and we distinguish them from *appointment slots* that are consecutive timeslots for a station (chair or bed) in the template. Unless otherwise stated, we refer to drug administration appointments simply as *appointments*.

**Fig. 4:**
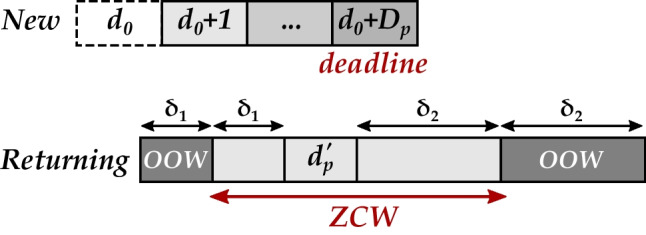
Days that we include in our heuristic for deciding the next appointment day of *new* and *returning* patients

Every day, a number of requests arrive at the clinic for making appointments for patients. We divide the appointment requests into two groups of *new* and *returning* patients. New patients are not yet registered in the system, i.e., no appointment has ever been made for them. Returning patients are already registered in the system, and at least one appointment has been made for them. No appointment is made for the current day *d*_0_: the day the request arrives. As illustrated in Fig. 4, appointments for new patients are expected to be scheduled from the day after the request arrives until a *deadline*. Appointments for returning patients are expected to be scheduled as many as possible within their ZCWs and never more than the tolerances out of the ZCWs (Fig. 4). If the ZCW falls on a weekend—or a holiday for that matter—and the clinic is closed, then at the time of request, the desired day can be changed to Monday or Friday with *δ*_1,*p*_ = *δ*_2,*p*_ = 0 instead of their default values in the patient’s treatment protocol.

Requests from new patients arrive randomly at any timeslot during the opening hours and from returning patients at the beginning of their current-day appointments. Appointments canceled due to unsatisfactory test results get rescheduled for seven days later with *δ*_1,*p*_ = *δ*_2,*p*_ = 0. The request for rescheduling arrives at the beginning of the canceled appointment. If an appointment gets canceled, its subsequent planned days are also *implicitly* postponed. Hence, when a request arrives, only the next appointment is scheduled. The remaining appointments in the patient’s prescribed treatment protocol are not scheduled because of the possibility of cancelation of the next appointment or change of treatment, which render the appointments scheduled farther in the future, invalid. Before their planned overall treatments begin, patients and their family members are briefed about the importance of adhering to the appointment schedules for treatment effectiveness and the limited availability of resources. Hence, there is a negligible number of no-shows and late arrivals [11].

**Fig. 5:**
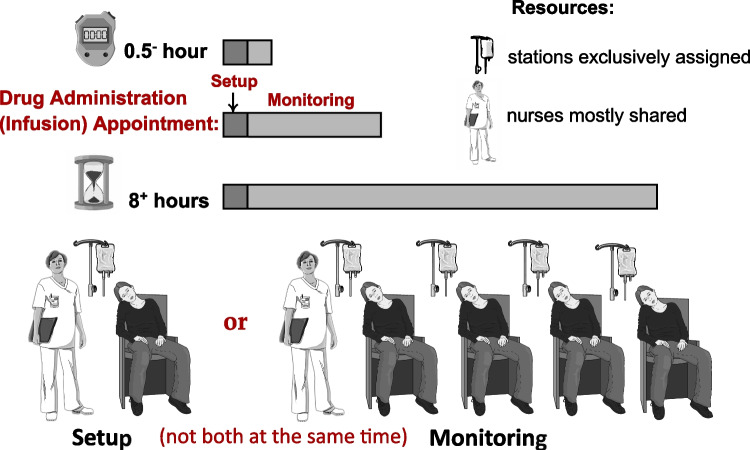
General arrangements of drug administration appointments in outpatient chemotherapy

During coffee and lunch breaks, the number of available nurses *N* drops to half. Hard constraints are enforced on the two nursing tasks: *setting up* and *monitoring*. During the first timeslot of every appointment, one nurse must be fully present for preparing the patient and setting up the station. During the remainder of the appointment, a nurse who is neither on a coffee or lunch break nor busy with a setup, monitors the infusion progress. A nurse can simultaneously monitor up to *M* patients. Every nurse is allowed to carry out both setting up and monitoring tasks, but not simultaneously in the same timeslot. These hard constraints on nursing are illustrated in Fig. 5. In our fixed template heuristic, these hard constraints are already incorporated into the given template, i.e., in its design. The first timeslot of each slot in the template is foreseen for setup. Hence, because of the hard constraints, when booking, the appointment must be placed at the beginning of a template slot. The notation used in the models is listed in Table 1.

**Table 1:** Table of notation for the proposed models

Notation	Description
	***Indexes:***
*d*	index for the day in the appointment scheduling BIP (heuristic)
*n*	index for the nurse in the task scheduling MIP
*p*	index for the patient in the appointment scheduling BIP (heuristic) and the task scheduling MIP
*s*	index for the slot in the appointment scheduling BIP (heuristic)
*t*	index for the timeslot in the task scheduling MIP
	***Input Parameters:***
$\pmb {A}_{N \times T^{\prime }}$	constant binary matrix indicating nurse availability throughout the (extended) opening hours
$\mathcal {E}$	set of last slots at all stations in the appointment template (not *z*-app.): end-slots (light-gray)
*K*	number of stations in the template
*M*	number of patients that can be simultaneously monitored by one nurse
*N*	total number of nurses (full-time equivalent) accounted for in the template
*N*_*t*_	number of nurses available for the setting up or monitoring tasks in timeslot *t*
*S*	number of slots in the template
*T*	number of timeslots in the regular opening hours in a vacant template
*a*_*n*,*t*_	flag (binary parameter): 1 if nurse *n* available to work in timeslot *t*
*l*_*s*_	duration of slot *s*
$\hat {\tau }_{s}$	first timeslot of slot *s* in the template
	***Simulation and Other Random Quantities:***
*A*	random variable for the overall number of installments (appointments) in a patient’s regimen
*C*	random variable for the number of cycles in a patient’s regimen
*D*	random variable for the number of planned days in a cycle of a patient’s regimen
*D*_*p*_	deadline for the first appointment of new patient *p* after receiving the request
*D*^*plan*^	number of days in the simulation, including both transient and steady-state periods
*H*	random variable for the cycle duration of a patient’s regimen
$\mathcal {N}$	set of requests from new patients being considered at a decision moment on the current day
$\mathcal {O}$	set of requests from returning patients being considered at a decision moment on the current day
$\mathcal {R}$	set of requests being considered at a decision moment on the current day: $\mathcal {R} = \mathcal {N} \cup \mathcal {O}$
$d^{\prime }_{p}$	desired (planned) day for the next appointment of returning patient *p*
$l^{\prime }_{p}$	required appointment duration for patient *p*
*δ*_1,*p*_, *δ*_2,*p*_	number of days before or after the planned day with negligible side effects or diminishing results
*λ*	average rate of the Poisson arrival process for new patients
*λ*_*high*_	new patient arrival rate corresponding to the service rate of the template: $\lambda _{high} \mathbb {E}(A) = S$
	***Decision Variables:***
*v*_*p*,*n*,*t*_	binary variable: 1 if nurse *n* takes care of patient *p* in timeslot *t*, and 0 otherwise
*x*_*p*,*d*,*s*_	binary variable: 1 if patient *p* booked for day *d* in slot *s*, 0 otherwise
*y*_*p*,*d*,*s*_	binary variable: 1 if patient *p* booked for day *d* in end-slot *s* by slot extension, 0 otherwise
*z*_*p*,*d*_	binary variable: 1 if patient *p* booked for day *d* by slot addition, 0 otherwise
*𝜖*_*n*_	extra amount of workload assigned to nurse *n* that is above the average workload of the day (Γ)
*η*_*p*,*n*,*t*_	binary variable: 1 if nurse *n* starts taking care of patient *p* in timeslot *t*
	***Intermediary Parameters:***
$\mathbbm{1}_{{\mathscr{S}}}$	the indicator function: 1 if statement ${\mathscr{S}}$ is *true*, and 0 otherwise
*B*_*i*_	constants (parameters) in the BIP to keep various penalties at different levels
*C*_*p*,*d*,*s*_	completion time of appointment *p* in slot *s* on day *d*
$\pmb {F}_{D^{plan} \times S}$	binary matrix indicating slot occupancy flags of templates on all days
*L*	an extremely large value
*OOW*_*p*,*d*_	number of days that day *d* is out-of-window for returning patient *p*
*d*_0_	current day on which decisions are made for appointments on future days
*d*_*H*_	last day considered in the BIP (heuristic), i.e., latest possible day among all patients in $\mathcal {R}$
*f*_*d*,*s*_	flag: 1 if slot *s* of day *d* is already booked for a patient and not available at the decision moment
$p \ntriangleright s$	priority patient *p* being booked in non-priority slot *s*
$s \ntriangleright p$	priority slot *s* being booked for non-priority patient *p*
Φ_*p*,*d*,*s*_	cost (penalty) of scheduling an *x*-appointment for patient *p* on day *d* in slot *s*
Ψ_*p*,*d*,*s*_	cost (penalty) of scheduling a *y*-appointment for patient *p* on day *d* in slot *s*
Θ_*p*,*d*_	cost (penalty) of scheduling a *z*-appointment for patient *p* on day *d*
*β*	weighted-sum parameter for the bicriterion nurse task scheduling MIP
	***Solution Quantities:***
*C*_*max*_	makespan of a day
$\pmb {G}_{P \times T^{\prime }}$	binary matrix: setup timeslots of appointments in a finalized online schedule
*P*	number of booked appointments in a finalized schedule
$T^{\prime }$	number of timeslots in the schedule of a day, possibly with overtime, i.e., $T^{\prime } > T$
*g*_*p*,*t*_	flag (binary parameter): 1 if the station setup for patient *p* is in timeslot *t* (finalized schedule)
*x*-app.	an appointment placed within a template slot
*y*-app.	an appointment placed in an end-slot by extending the slot
*z*-app.	an appointment placed after a filled end-slot
Γ	average workload (in equivalent number of station-timeslot monitors) per nurse on a day

## Online scheduling principles for using a fixed template

**Fig. 6:**
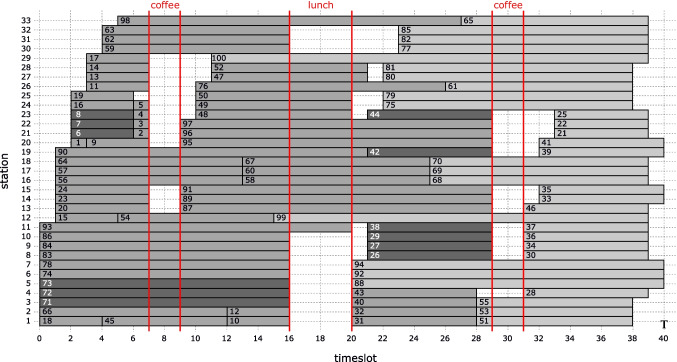
The 100-slot template (*S* = 100) used in simulating our heuristic (BIP model). The number at the beginning of each slot is its index (*s* = 1,⋯ , *S*). The vertical lines demarcate the coffee and lunch breaks. The end-slots of the *K* = 33 stations are light-gray. Twelve slots allocated as priority are dark-gray. The nursing capacity is *N* = 11 nurse full-time equivalent (FTE). Each nurse can monitor up to *M* = 4 patients or set up one station. The regular opening hours are *T* = 40 timeslots (ten hours)

In this section, we propose using a fixed template with a set of principles for the online scheduling of drug administration appointments in outpatient chemotherapy. The template is the fixed arrangement of *vacant* appointment slots shown in Fig. 6.

**Fig. 7:**
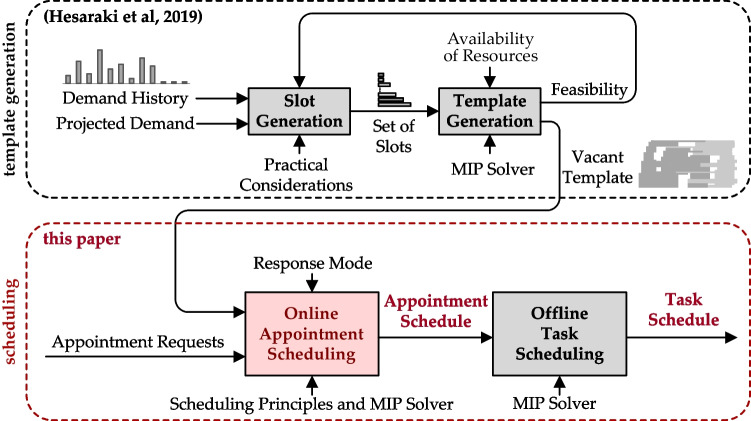
Template generation followed by online appointment scheduling and offline task scheduling

A high-level block diagram of the overall model that we consider for online scheduling of appointments is illustrated in Fig. 7. The template is generated based on the history of appointments (e.g., [12]). In this paper, we focus on the last two blocks.

The template is an input to our model. It is already designed subject to the setting up and monitoring constraints and based on minimizing a combination of flowtime and makespan. Requirements from the pharmacy or oncologists for starting some appointment slots of the template close to a given time or not starting some slots until a specific time are incorporated into the fixed template [12]. Those slots are highlighted in dark-gray in Fig. 6. The appointments of some patients should be scheduled with priority in those slots, which is indicated in their appointment requests. The template is used over a long horizon, e.g., one year. The basic ideas in our proposed heuristic are outlined in the following six scheduling principles for using the template:

- **P.1.** Schedule the appointments of new patients as soon as possible between the day after the request arrives and the deadline.
- **P.2.** Schedule the appointments of returning patients as many as possible in their ZCWs.
- **P.3.** Place priority appointments, as many as possible, in the slots allocated for them.
- **P.4.** Place as many appointments as possible in the template slots with as few as possible idle timeslots at the end of the booked slots.
- **P.5.** When there are no vacant slots long enough for an appointment, extend a vacant end-slot (colored light-gray in the template of Fig. 6) that would result in the earliest possible completion time, i.e., with as little as possible overtime after the default closing time (*T*).
- **P.6.** When there is no long enough vacant slot and all end-slots of the template are taken, as a last resort, add the appointment at the earliest possible time after an end-slot. For returning patients, these *additional* appointment slots are allowed only within the ZCWs.

We incorporate all six principles into the objective function cost coefficients of a BIP. In the model, the current day—the day that a request arrives for the next appointment—is denoted by *d*_0_. For every patient *p* being considered in the BIP, the required duration of the appointment is denoted by $l^{\prime }_{p}$. A new patient *p* can get the first appointment between the day after the request and a deadline: $d_{0}+1 \leqslant d \leqslant d_{0} + D_{p}$. For returning patient *p*, the desired (planned) day for the next appointment is denoted by $d_{p}^{\prime }$. The ZCW spans from *δ*_1,*p*_ days before to *δ*_2,*p*_ days after that desired day $d_{p}^{\prime }$.

On each day *d*, the template has *S* slots numbered *s* = 1,⋯ , *S*. Set $\mathcal {E}$ is the set of last slots at all stations. An attribute of the $\mathcal {E}$ slots is that there is the possibility to extend them for appointments longer than the slots without running into another slot. For $s \in \mathcal {E}$, overtime is incurred if $\hat {\tau }_{s}+l^{\prime }_{p}-1 > T$, where $\hat {\tau }_{s}$ is the first timeslot of slot *s* and *T* is the *regular* closing time of the clinic. The regular working hours of the clinic are *T* consecutive timeslots, *t* = 1,⋯, *T*, where timeslot *t* is the time interval (*t* − 1, *t*].

The set of requests being considered at a decision moment on the current day *d*_0_ is denoted by $\mathcal {R}$. Within that, the subsets of requests from new and returning patients are denoted by $\mathcal {N}$ and $\mathcal {O}$, respectively: $\mathcal {R} = \mathcal {N} \cup \mathcal {O}$. Day *d* is a day being considered for the appointment. Slot *s* is a slot of the template being considered for the appointment on that day. *l*_*s*_ is the duration of slot *s* measured in timeslots. The decision variables of the BIP are defined as follows: *x-appointments* (within the template):

$$x_{p,d,s}=\left\{\begin{array}{ll}1,&\;\mathrm{patient}\;p\;\mathrm{placed}\;\mathrm{on}\;\mathrm{day}\;d\mathit\;\mathrm{within}\;\mathrm{slot}\;s\;\\0,&\;\mathrm{otherwise}\end{array}\right.$$

*y-appointments* (extending the template):

$$y_{p,d,s}=\left\{\begin{array}{ll}1,&\;\mathrm{patients}\;p\;\mathrm{placed}\;\mathrm{on}\;\mathrm{day}\;d\;\mathrm{in}\;\mathrm a\;\mathrm{shorter}\;\mathrm{than}\\&\;\mathrm{needed}\;\mathrm{end}\; \mathrm{slot}\;s\;\in\mathcal E\;\mathrm{by}\;\mathrm{slot}\;\mathrm{extension}\;\\0,&\;\mathrm{otherwise}\end{array}\right.$$

*z-appointments* (adding to the template):

$$z_{p,d}=\left\{\begin{array}{ll}1,&\mathrm{patient}\;p\;\mathrm{placed}\;\mathrm{on}\;\mathrm{day}\;d\;\mathrm{after}\;\mathrm{a}\;\mathrm{booked}\;\mathrm{end}\;\mathrm{slot}\\&\mathrm{appointment}:\mathrm{before}\;\mathrm{the}\;\mathrm{deadline}\;D_p\;\mathrm{or}\;\mathrm{in}\;\mathrm{the}\;\mathrm{ZCW}\;\\0,&\mathrm{otherwise}\end{array}\right.$$

**Fig. 8:**
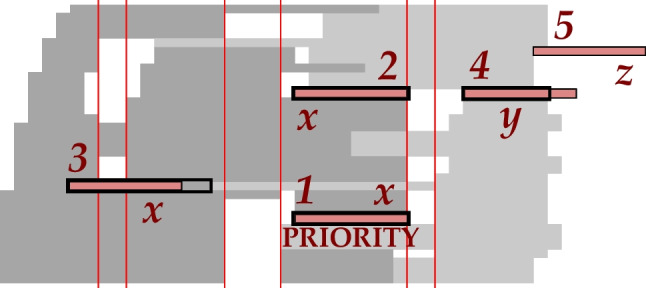
Slot selection in descending order of preference: priority (*x*-appointment), perfect match (*x*-appointment), least idle time (*x*-appointment), least overtime (*y*-appointment), slot addition (*z*-appointment)

Note that the end-slots are not exclusively used with extension. They can accommodate either *x*-appointments or *y*-appointments. The preferences given to slots when placing an appointment is shown in Fig. 8, and they are in the following order: 1) Preferably, priority appointments are booked in their allocated slots. 2) Ideally, a slot that matches the desired appointment duration will be used. 3) If no perfectly matching slot is available, a slot longer than the desired appointment will be used. However, preference is given to a slot that would result in fewer idle timeslots after the appointment ends. 4) If the appointment does not fit into any slot, it can be booked in a vacant end-slot by extending that slot to the appointment duration. Preference is given to a vacant end-slot that results in the earliest completion time. 5) If all end-slots are also filled, the appointment is made after an end-slot that would result in the least amount of overtime.

The number of days that day *d* is out-of-window for returning patient *p* is indicated by the parameter *OOW*_*p*,*d*_:

$$OOW_{p,d} = \left\{\begin{array}{ll} d_{p}^{\prime}-\delta_{1,p}-d \ , &\quad d<d_{p}^{\prime}-\delta_{1,p} \\ 0 \ , & \quad d_{p}^{\prime}-\delta_{1,p} \leqslant d \leqslant d_{p}^{\prime}+\delta_{2,p} \\ d-d_{p}^{\prime}-\delta_{2,p} \ , & \quad d>d_{p}^{\prime}+\delta_{2,p} \end{array}\right.$$

The decision costs associated with *x*_*p*,*d*,*s*_, *y*_*p*,*d*,*s*_, and *z*_*p*,*d*_ are indicated by the cost coefficients Φ_*p*,*d*,*s*_, Ψ_*p*,*d*,*s*_, and Θ_*p*,*d*_, in Eqs. (1), (2), and (3), respectively. This is one example for realizing the six scheduling principles; several adjustments may be possible for constructing the BIP objective function coefficients around the desired principles.

1 $$\begin{array}{@{}rcl@{}} {\Phi}_{p,d,s} = \left\{\begin{array}{lcc} 2^{B_{1}+d-d_{0}} + \mathbbm{1}_{(p \ntriangleright s)}2^{B_{3}} + \mathbbm{1}_{(s \ntriangleright p)}2^{B_{4}} + & p \in \mathcal{N} \enspace \wedge \enspace f_{d,s}=0 \enspace \wedge \enspace l_{s}\geqslant l^{\prime}_{p} \enspace \wedge \\ \qquad \mathbbm{1}_{(l_{s} > l_{p}^{\prime})}\Big \lceil 2^{B_{5}+(l_{s}-l^{\prime}_{p})/2} \Big \rceil + \hat{\tau}_{s} \ , &\!\!\!\!\!\!\!\!\! \!\!\!\!\!\!\!\!\!\!\!\!\!\!\!\!\!\!\! d_{0}+1 \leqslant d \leqslant d_{0} + D_{p} \enspace\\ \mathbbm{1}_{(OOW_{p,d} > 0)}2^{B_{2}+OOW_{p,d}} + \mathbbm{1}_{(p \ntriangleright s)}2^{B_{3}} + \mathbbm{1}_{(s \ntriangleright p)}2^{B_{4}} + & \quad\!\!\!\!\! p \in \mathcal{O} \enspace \wedge \quad f_{d,s}=0 \enspace \wedge \enspace l_{s}\geqslant l^{\prime}_{p} \enspace \wedge \\ \qquad \mathbbm{1}_{(l_{s} > l_{p}^{\prime})}\Big \lceil 2^{B_{5}+(l_{s}-l^{\prime}_{p})/2} \Big \rceil + \hat{\tau}_{s} \ , & \quad \max\{d_{0}+1,d_{p}^{\prime}-2\delta_{1,p}\} \leqslant d \leqslant d_{p}^{\prime}+2\delta_{2,p} \enspace \\ L \ , & \!\!\!\!\!\!\!\!\!\!\!\!\!\!\!\!\!\!\!\!\!\!\!\!\!\!\!\!\!\!\!\!\!\!\!\!\!\!\!\!\!\!\!\!\!\!\!\!\!\!\!\!\!\!\!\!\!\!\!\!\!\!\!\!\! \mathrm{otherwise} \end{array}\right. \end{array}$$

2 $$\begin{array}{@{}rcl@{}} {\Psi}_{p,d,s} = \left\{\begin{array}{lcc} 2^{B_{1}+d-d_{0}} + \mathbbm{1}_{(p \ntriangleright s)}2^{B_{3}} + \mathbbm{1}_{(s \ntriangleright p)}2^{B_{4}} + & \quad p \in \mathcal{N} \enspace \wedge \enspace s \in \mathcal{E} \enspace \wedge \enspace f_{d,s} = 0 \enspace \wedge \enspace l_{s}<l^{\prime}_{p} \enspace \wedge \\ \qquad 2^{B_{6}} + \mathbbm{1}_{(C_{p,d,s}>T)}2^{C_{p,d,s}-T} \ , & \!\!\!\!\!\!\!\!\!\!\!\!\!\!\!\!\!\!\!\!\!\!\!\!\!\!\!\!\!\!\!\!\!\!\!\!\!\!\!\!\!\!\!\!\!\!\!\!\! d_{0}+1 \leqslant d \leqslant d_{0} + D_{p} \\ \mathbbm{1}_{(OOW_{p,d} > 0)}2^{B_{2}+OOW_{p,d}} + \mathbbm{1}_{(p \ntriangleright s)}2^{B_{3}} + \mathbbm{1}_{(s \ntriangleright p)}2^{B_{4}} + & \quad p \in \mathcal{O} \enspace \wedge \enspace s \in \mathcal{E} \enspace \wedge \enspace f_{d,s} = 0 \enspace \wedge \enspace l_{s}<l^{\prime}_{p} \enspace \wedge \\ \qquad 2^{B_{6}} + \mathbbm{1}_{(C_{p,d,s}>T)}2^{C_{p,d,s}-T} \ , & \quad \max\{d_{0}+1,d_{p}^{\prime}-2\delta_{1,p}\} \leqslant d \leqslant d_{p}^{\prime}+2\delta_{2,p} \enspace \\ L \ , & \!\!\!\!\!\!\!\!\!\!\!\!\!\!\!\!\!\!\!\!\!\!\!\!\!\!\!\!\!\!\!\!\!\!\!\!\!\!\!\!\!\!\!\!\!\!\!\!\!\!\!\!\!\!\!\!\!\!\!\!\!\!\!\!\!\!\!\!\!\!\!\!\!\!\!\!\!\! \mathrm{otherwise} \end{array}\right. \end{array}$$

3 $$\begin{array}{@{}rcl@{}} {\Theta}_{p,d} & = \left\{\begin{array}{lcc} L/4 \ , \quad [ \ p \in \mathcal{N} \enspace \wedge \enspace d_{0}+1 \leqslant d \leqslant d_{0} + D_{p} \ ] \enspace \vee \\ \quad\quad \quad [ \ p \in \mathcal{O} \enspace \wedge \enspace \max\{d_{0}+1,d_{p}^{\prime}-\delta_{1,p}\} \leqslant d \leqslant d_{p}^{\prime}+\delta_{2,p} \ ] \ \\ L \ , \quad \quad \mathrm{otherwise} \end{array}\right. \end{array}$$

Φ_*p*,*d*,*s*_ helps to place *x*-appointments into the template slots with as little idle time as possible at the end of the slots. Ψ_*p*,*d*,*s*_ helps to place *y*-appointments in end-slots by extension with as little overtime as possible. Θ_*p*,*d*_ helps to place *z*-appointments after the end-slots as a last resort. The values for the constants *B*_*i*_ should be chosen based on the numerical settings of the problem, such as the latest deadline $\max \limits (D_{p})$, the greatest tolerance value $\max \limits \{\delta _{1,p},\delta _{2,p}\}$, and also the greatest integer value before numerical overflow in the computation environment. The goal is to have the various types of penalties at different levels according to our preferences for the six scheduling principles, as we explain in this section.

The flag parameter *f*_*d*,*s*_ is 0 when slot *s* is vacant and 1 when it is already booked for a patient on day *d*. These flags are kept in the binary matrix $\mathbf {F}_{D^{plan} \times S}$, which is updated whenever the BIP is solved or an appointment gets canceled due to unsatisfactory test results. *L* is an extremely large value. When placing patient *p* in slot *s* on day *d*, i.e., *x*_*p*,*d*,*s*_ = 1 or *y*_*p*,*d*,*s*_ = 1, the slot must be vacant: *f*_*d*,*s*_ = 0. Otherwise, the penalty associated with that placement will be extremely large (*L*) to avoid it.

The cost coefficients in Eqs. (1) and (2) are the sum of day-penalties and slot-penalties. We formulate the BIP to have a *significantly* greater penalty for one more day waiting for new patients, one more day out-of-window for returning patients, one more station-timeslot overtime, and one more idle station-timeslot. To that end, we use *exponential* increments in the penalties. Besides these criteria, we also include the priority of patients and slots, and the start time (first timeslot) of slots. In order to have our preferences met among these multiple criteria based on their importance for the bottom line of the service, i.e., timely drug administration for an effective treatment, we put each type of penalty at a numerical level in relation to the others by using the constants *B*_*i*_ in Eqs. (1) and (2). We use exponents of 2 for the values of the penalty coefficients to avoid numerical overflow while keeping the penalties in non-overlapping ranges of values.

In the cost coefficients, we make a distinction between *new* and *returning* patients. The terms that include *B*_1_ and *B*_2_ correspond to the day-deviation penalties of *new* and *returning* patients, respectively. The most important criterion, as we previously quoted [7], is the timeliness of the treatment: little wait for new patients and ZCW appointments for returning patients. Enforcing the deadlines as hard constraints takes care of that requirement for new patients. Note that deadlines can be notably longer than the zero-cost windows, and they apply to notably fewer appointments (only the first appointments). Hence, for returning patients, we use soft constraints (penalties) but with the highest level of penalty in the BIP to have as few OOW appointments for them as possible.

Whenever a priority-deviation (a special case of slot-penalty) would incur, one of two fixed-value penalties would be enforced: one if it involves a priority patient and the other if it involves a priority slot. However, since the day of appointment is more important for the patient’s treatment than the possible consequences of not placing the appointment in a preferentially allocated slot, the priority penalties should be at a lower level than the OOW penalties. Otherwise, out-of-window placement will incur in favor of priority. $\mathbbm {1}_{{\mathscr{S}}}$ is the indicator function: 1 if statement ${\mathscr{S}}$ is *true*, and 0 otherwise. $p \ntriangleright s$ denotes priority patient *p* being booked in non-priority slot *s*, and $s \ntriangleright p$ denotes priority slot *s* being booked for non-priority patient *p*. The former is more critical as it violates a placement priority and must be given a greater penalty than the latter, which only pertains to the potential loss of opportunity in the future for the placement of a priority patient. The terms with *B*_3_ and *B*_4_ correspond to these two priority-deviations.

We consider overtime more undesirable than idle time as the clinic incurs further cost besides the decline in nurse and patient satisfaction. The terms with *B*_5_ and *B*_6_ correspond to idle time and overtime, respectively. For placements within slots, *x*_*p*,*d*,*s*_ = 1 with Φ_*p*,*d*,*s*_ < *L*, it must hold that $l_{s}\geqslant l_{p}^{\prime }$. Otherwise, the penalty associated with such placements will be extremely large (*L*) to avoid them. Idle time incurs when $l_{s} > l_{p}^{\prime }$. To use less of the numerical range before overflow in the computation environment, we double the idle time penalty for every two further idle station-timeslots, rather than every further idle station-timeslot, incurring at the end of the booked slot. We include *B*_5_ to keep the idle time penalty at a greater value than the start time penalty $\hat {\tau }_{s}$, which is there to get earlier slots filled first when everything else is equal. Including $\hat {\tau }_{s}$ is in alignment with patients’ general preference to have infusions earlier during the day. For slot extension, *y*_*p*,*d*,*s*_ = 1 with Ψ_*p*,*d*,*s*_ < *L*, the slot must be an end-slot shorter than the desired appointment duration: $s \in \mathcal {E}$ and $l_{s} < l_{p}^{\prime }$. Otherwise, the penalty associated with that placement will be extremely large to avoid that *y*-appointment. $C_{p,d,s}=\hat {\tau }_{s}+l_{p}^{\prime }-1$ is the completion time of appointment *p* in slot *s* on day *d*. A *y*-appointment has the possibility of running overtime, though it may be shorter than half the idle time of a feasible *x*-appointment for the same request. We therefore use the constant *B*_6_ to make a *y*-appointment less desirable (with a higher penalty) than an *x*-appointment. *B*_6_ must be just greater than the maximum penalty for idle time.

It should be noted that we attempt to effectively and efficiently use the allocated service capacity of the template without resorting to *z*-appointments as much as possible. The number of *z*-appointments should be viewed as the most important indicator of performance. The greatest penalty for feasible solutions in the BIP is *L*/4 for *z*-appointments. A high number of *z*-appointments implies the allocated capacity is insufficient for demand. Thus, the order of *importance* that we consider for the various types of penalty is as follows: 
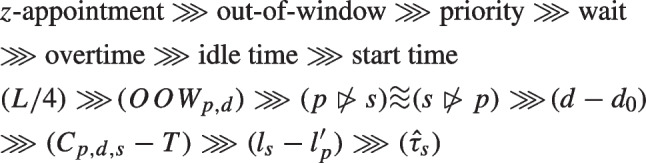


The BIP can be applied in two different modes of responding to appointment requests: *immediately* and *daily*. In immediately scheduling, the decisions about requests are made one-by-one in the moment a request arrives. In this mode, we always have $|\mathcal {R}|=1$. In daily scheduling, the decision moments are every 24 hours. The requests that arrive on the current day *d*_0_ are collected and then decided for at the end of the day, all at once. Both the immediately and daily use of the scheduling BIP are online scheduling because in neither case, the complete set of jobs to be scheduled for any day *d* in the future is known a priori. In particular, no exact information about the total appointments on a day is known a priori. However, in daily scheduling, more information is available at the decision moment.

The last day that has to be included in the BIP is the latest possible day among all patients in $\mathcal {R}$:

4 $$d_{H} = \max\bigg\{\ d_{0} + \underset{p \in \mathcal{N}}{\max} (D_{p}) \ , \ \underset{p \in \mathcal{O}}{\max} (d_{p}^{\prime}+2\delta_{2,p}) \bigg\}$$

**Fig. 9:**
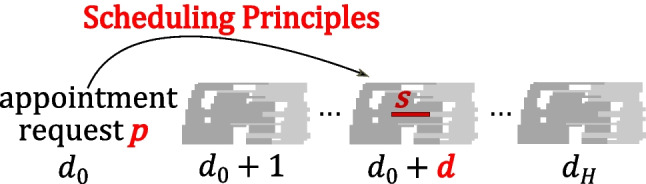
For both new and returning patients, the scheduling principles determine the days and slots of appointments over *d*_*H*_ − *d*_0_ days after the request is received on the current day *d*_0_

Thus, as shown in Fig. 9, the scheduling principles incorporated into the BIP determine the appointment day and the appointment slot on that day within *d*_*H*_ − *d*_0_ days after the request is received on day *d*_0_.

The BIP formulation of the scheduling principles is as follows:

5a $$\begin{array}{@{}rcl@{}} &\min& \sum\limits_{p \in \mathcal{R}} \ \sum\limits_{d=d_{0}+1}^{d_{H}}\bigg[ \sum\limits_{s=1}^{S} \Big({\Phi}_{p,d,s} x_{p,d,s} + {\Psi}_{p,d,s} y_{p,d,s} \Big)\\ &&+ {\Theta}_{p,d} z_{p,d} \bigg] \end{array}$$

5b $$\begin{array}{@{}rcl@{}} &{s.t.} & \sum\limits_{d=d_{0}+1}^{d_{H}}\bigg[ \sum\limits_{s=1}^{S} \big(x_{p,d,s} + y_{p,d,s} \big) + z_{p,d} \bigg]= 1 \\ &&{\ \ for \ \ } p \in\mathcal{R} \qquad \mathrm{(one\ per\ patient)} \end{array}$$

5c $$\begin{array}{@{}rcl@{}} & &\sum\limits_{p \in \mathcal{R}} \big(x_{p,d,s} + y_{p,d,s} \big) \leqslant 1 {\ \ for \ \ } \\ && d=d_{0}+1, \cdots, d_{H} {\ \ and \ \ } s=1, \cdots, S \\&& \mathrm{(at\ most\ one\ per\ slot)} \end{array}$$

The objective function (5a) incorporates the scheduling principles in the coefficients. Constraints (5b) are for having exactly one appointment per patient. Constraints (5c) are intended to prevent assigning a slot to more than one patient.

The variables *z*_*p*,*d*_ allow to properly schedule appointments as a last resort when there is no slot vacant for a feasible placement. Without that provision, the solver may give an infeasible solution for the BIP. When *z*_*p*,*d*_ = 1, patient *p* is treated after a booked end-slot on a day before the deadline or within the ZCW. The total number of booked *z*-appointments is an indicator of the amount of demand overload for the allocated capacity in the template. It can be used for adjusting the template in the next tactical plan of the clinic.

On the current day *d*_0_, an appointment may get canceled due to undesirable test results, with probability $\mathbb {P}(it {cancel})$. It is then rescheduled for a certain number of days later. The remaining planned days in the treatment protocol and the resting periods among them will continue from that rescheduled appointment.

## Offline task scheduling

The template is designed based on *N*_*t*_ nurses being available for drug administration in timeslot *t*, e.g., *N* nurses working the whole day except for their coffee and lunch breaks, during which, only half of the nurses are working. In this section, we present a mixed integer program (MIP) adopted from a special case of the integrated offline scheduling model of [13] for assigning the setup and monitoring tasks to the nurses. The objective is to have equal workload distributed among the nurses and as few nurse changes during appointments as possible. With fewer nurse changes, the service is safer and less confusing for patients and nurses. The nurse task scheduling MIP is formulated as follows over the extended working hours ($t = 1, \cdots , T^{\prime }$) in the finalized appointment schedule:

6a $$\begin{array}{@{}rcl@{}} &\min & \quad\Bigg [ \beta \bigg (\sum\limits_{p=1}^{P}\sum\limits_{n=1}^{N}\sum\limits_{t=1}^{T^{\prime}} \eta_{p,n,t} \bigg ) + (1-\beta) \sum\limits_{n=1}^{N} \epsilon_{n} \Bigg ] \end{array}$$

6b $$\begin{array}{@{}rcl@{}} &{s.t.} & \quad \sum\limits_{p=1}^{P} \big[ 1 + (M-1)g_{p,t} \big] v_{p,n,t} \leqslant M a_{n,t} \quad ; \quad\\ && \forall n, \forall t \quad {(\mathrm{nursing\ capacity})} \end{array}$$

6c $$\begin{array}{@{}rcl@{}} & & \sum\limits_{n=1}^{N} v_{p,n,t} = \sum\limits_{i=0}^{\min \{t-1, l^{\prime}_{p}-1\}} g_{p,t-i} \quad ; \quad \forall p, \forall t \quad \\&&{(\mathrm{appointment\ period})} \end{array}$$

6d $$\begin{array}{@{}rcl@{}} & & \sum\limits_{n=1}^{N} v_{p,n,t} \leqslant 1 \quad ; \quad \forall p, \forall t \quad {(\mathrm{one\ nurse})} \end{array}$$

6e $$\begin{array}{@{}rcl@{}} & & \Bigg \{ \sum\limits_{p=1}^{P}\sum\limits_{t=1}^{T^{\prime}} \big[ 1 + (M-1)g_{p,t} \big] v_{p,n,t} \Bigg \} - {\Gamma} \leqslant \epsilon_{n} \quad ; \quad \\ &&\forall n \quad {(\mathrm{workload})} \end{array}$$

6f $$\begin{array}{@{}rcl@{}} & & v_{p,n,t+1} - v_{p,n,t} \leqslant \eta_{p,n,t+1} \quad ; \quad \\&& \forall p, \forall n; \ t=1,\cdots, T^{\prime}-1 \quad \\ && {(\mathrm{nurse\ change})} \end{array}$$

$T^{\prime }$ is equal to *C*_*max*_, the makespan of the finalized appointment schedule.

The binary variable *v*_*p*,*n*,*t*_ is 1 when nurse *n* takes care of patient *p* in timeslot *t* (either setting up or monitoring), and 0 otherwise. The binary variable *η*_*p*,*n*,*t*_ is 1 when nurse *n* starts taking care of patient *p* in timeslot *t*, and 0 otherwise. The variable $\epsilon _{n} \in \mathbb {R}^{+}$ is the extra amount of workload assigned to nurse *n* above the average workload (Γ) of the day, calculated based on the total nurse FTE on the day: ${\Gamma } = (1/N)[(M-1)P+\sum \nolimits _{p=1}^{P}l^{\prime }_{p}]$.

Since a setup consumes the nursing capacity of *M* patients being monitored, in Γ, we consider the workload of a setup equivalent to that of *M* patients being monitored, and we express the nurses’ workloads in terms of the equivalent number of patients being monitored. For simplicity, we refer to the counts of these two tasks as *setups* and *monitors*. The availability of each nurse on the intended day is indicated in a constant binary matrix $\mathbf {A}_{N \times T^{\prime }} = [a_{n,t}]_{N \times T^{\prime }}$, where the element *a*_*n*,*t*_ is 1 when nurse *n* is available for setting up or monitoring in timeslot *t*, and 0 otherwise. It must also conform to *N*_*t*_: $\sum \nolimits _{n=1}^{N} a_{n,t} \geqslant N_{t}, \forall t$, not fewer than the number considered in the template design.

After the template is filled and the schedule is finalized with *P* patients booked in it, the appointments are numbered as *p* = 1,⋯ ,*P*. These may include, *z*-appointments and *y*-appointments besides the perfectly matched or partially idle *x*-appointment slots in the template. Thus, the setup timeslot of each appointment is known in a constant binary matrix $\mathbf {G}_{P \times T^{\prime }} = [g_{p,t}]_{P \times T^{\prime }}$, where the element *g*_*p*,*t*_ is 1 when *setup* takes place for appointment *p* in timeslot *t*, and 0 otherwise.

Every setup in $t \geqslant 2$ sets *η*_*p*,*n*,*t*_ equal to 1, although a setup is not an actual nurse change for the patient. At *t* = 1, all *N*_1_ nurses are available for setup. However, since the time index does not include *t* = 0, the *N*_1_ setups at *t* = 1, do not set *η*_*p*,1,*t*_ equal to 1. Hence, after solving the MIP model, the number of nurse changes during appointments can be calculated as $(\sum \nolimits _{p=1}^{P}\sum \nolimits _{n=1}^{N}\sum \nolimits _{t=1}^{T^{\prime }} \eta _{p,n,t} ) - (P-N_{1})$.

**Fig. 10:**
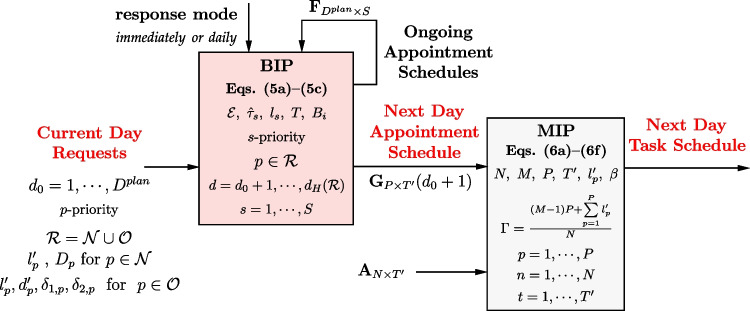
Resource allocation: online appointment scheduling followed by offline task scheduling

The procedure for online appointment scheduling followed by offline nurse task scheduling is shown in Fig. 10. Since some appointments may be scheduled for the day after the current day, the task schedule of the next day is generated at the end of the current day, i.e., when no more appointments will be added to the schedule.

## Numerical illustration

In this section, we use simulation to demonstrate how our proposed heuristic performs for various key indicators considered for multiple stakeholders. The computations of our numerical experiments were carried out on a 64-bit computer with Windows 10, Intel processor i7-9700 (3.00 GHz), and 32 GB of RAM. For solving the BIP and MIP models, we used Gurobi 9.5.1 in the Julia programming language, using its mathematical programming package JuMP [6].

There is no natural end-point that determines the length of a simulation run in our model. Hence, we analyze the steady-state performance of the BIP heuristic applied to a stylized clinic using the *batch means method* [28] with a long single run for each of 20 different cases (combinations of the arrival rate, cancelation, and the response mode) outlined in Table 2. In each long run, there are 30 batches of length 365 samples (days) after the transient period. Thus, the heuristic is simulated over 11,094 days.

**Fig. 11:**
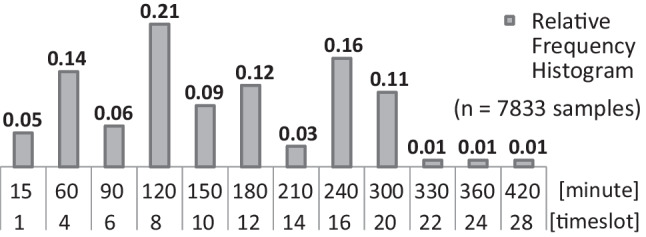
Categorical distribution of appointment durations among patients—adopted from [12]

The values we use for the treatments (patterns of planned days, cycle lengths, number of cycles, and ZCWs) conform to those reported in the literature [8, 11, 18, 20, 31, 34]. Since we independently combine their random values as to what the specific treatment details are for each patient when generating the *future event list* at the beginning of the simulation, it resembles the diversity of chemotherapy treatment protocols (regimens). To generate the future event list, we need to determine *λ*_*high*_, which depends on the expected number of overall treatment appointments per patient. Let *H*, *D*, *C*, and *A* be the random variables for the duration of a cycle, the number of planned days per cycle, the number of cycles, and the overall number of appointments in a patient’s treatment. Because of independence, $\mathbb {E}(A) = \mathbb {E}(D) \mathbb {E}(C)$. In the following, we lay out the simulation settings:

- Each patient has an *appointment duration* throughout the overall treatment that is drawn from the categorical distribution of Fig. 11. The template of Fig. 6 is also based on this distribution, which is derived from the appointment data of a hospital in The Netherlands. The template has *S* = 100 slots with the number of slots for each duration conforming to the distribution in Fig. 11. The slots are arranged among *K* = 33 stations and the template is designed for *N* = 11 nurses (11 × 1.0 FTE) during the day to set up one station or monitor up to *M* = 4 patients in each timeslot, if not on a break. The regular opening hours of the clinic without overtime is from 8:00 to 18:00 hrs. Each timeslot is 15 minutes, hence, *T* = 40. The slot arrangement in the template is optimized for the least flowtime at the minimum makespan within the opening hours [12].
- Each patient is randomly labeled to have one of seven *patterns* of planned days for all cycles: {1}, {1, 2}, {1, 11}, {1, 4, 9}, {1, 6, 11}, {1, 8, 15}, and {1, 2, 3, 4, 5, 8, 15} with probabilities 0.20, 0.20, 0.20, 0.14, 0.12, 0.12, and 0.02, respectively. Thus, $\mathbb {E}(D) = 1\times 0.20 + 2\times (0.20 + 0.20) + 3\times (0.14 + 0.12 + 0.12) + 7\times 0.02 = 2.28$ days per cycle.
- Each patient is randomly labeled to have one of four *number of cycles* 4, 6, 7, and 8 with probabilities 0.60, 0.25, 0.10, and 0.05, respectively. Thus, $\mathbb {E}(C) = 4\times 0.60 + 6\times 0.25 + 7\times 0.10 + 8\times 0.05 = 5.00$ cycles.
- There is an expected number of $\mathbb {E}(A) = 2.28 \times 5 = 11.40$ total appointments per patient. *New patient* arrival is simulated by a *Poisson process* with an average rate of *λ* = 6.0,6.5,7.0,8.0, and 8.4 patients per day, and $\lambda _{high} = S / \mathbb {E}(A) = 100 / 11.4 = 8.77$ patients arriving per day corresponds to the template capacity (new patient service rate). Thus, the simulated average arrival rates correspond to 68%, 74%, 80%, 91%, and 96% of the service rate allocated in the template: 8.77 new patients per day.
- The *instant* (timeslot) of a request is *uniformly* distributed over *t* = 1,⋯ , *T* for *new patients*. For *returning patients*, it is the *setup* timeslot of the current appointment. The time of request is only needed in simulating *immediately* scheduling.
- Each new patient has a maximum allowed indirect waiting time of three or seven days after making the initial request. Patients are randomly labeled with one of the two *deadlines D*_*p*_ = 3 and 7 with probabilities 0.15 and 0.85, respectively. Hence, the mean deadline is 3 × 0.15 + 7 × 0.85 = 6.4 days.
- The twelve dark-gray slots in the template of Fig. 6 are *preferentially* allocated for *priority* patients of the same appointment durations. The patients of those durations are randomly labeled priority with a probability equal to the ratio of priority slots to total slots of the corresponding duration: 3/14, 6/21, and 3/16.
- Each patient is randomly labeled to have one of four equally likely *ZCWs*: (*δ*_1,*p*_, *δ*_2,*p*_) = (0, 0), (1, 1), (1, 2), and (2, 2).
- Each patient is randomly labeled to have one of three *cycle-lengths*, 21, 28, and 42 days with probabilities 0.3, 0.5, and 0.2, respectively. Thus, $\mathbb {E}(H) = 21\times 0.3 + 28\times 0.5 + 42\times 0.2 = 28.7$ days.
- The deadline, priority, appointment duration, day-pattern, ZCW, cycle length, and number of cycles are randomly labeled independent of each other.
- Masselink et al. [24] report that for 8*%* of appointments the test results were unsatisfactory. In our simulation, each *returning* appointment may get canceled because of unsatisfactory test results with probability 0.1, which results in a rescheduling request for seven days later with (*δ*_1,*p*_, *δ*_2,*p*_) = (0, 0), regardless of the values of *δ*_1,*p*_ and *δ*_2,*p*_ for the treatment. In the numerical experiment, however, we also repeat each simulation case without cancelation to illustrate its impact on the key performance indicators (KPIs), which are measured quantities that show how well the heuristic works for various stakeholders.

**Fig. 12:**
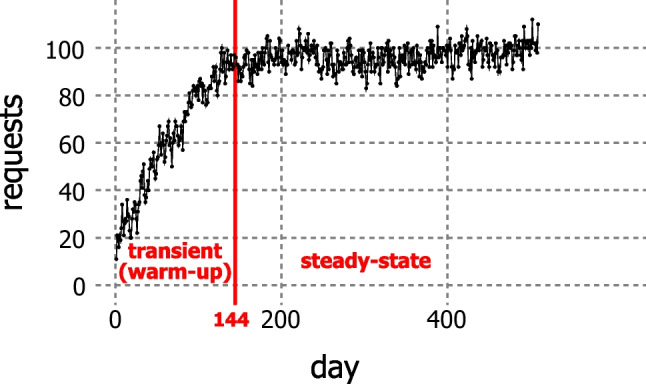
The number of requests builds up during the transient period until it settles at a steady level

Our simulations begin with an empty clinic (cold start). With the above settings, the expected transient (warm-up) period is $\mathbb {E}(transient) = \mathbb {E}(C) \mathbb {E}(H) = 5 \ {[cycle]} \times 28.7 \ {[day/cycle]} \approx 144$ days. Figure 12 shows the number of requests building-up in the transient period until it settles at a steady level. The goal for simulation is to see how the KPIs are affected in different settings in response to various appointment requests. We determine the average values of the KPIs over 10,950 days in the steady-state period. The simulated online scheduling starts on day *d*_0_ = 1 and ends on day *d*_0_ = *D*^*plan*^ = 144 + 30 × 365 = 11,094. The longest possible treatment period in our simulation is 336 days (eight 42-day cycles). Even if two appointments are postponed seven days each, the treatment (350 days) will still be shorter than 365 days, the length of a simulation batch. Hence, the batches can be considered independent or to have low correlation. On day *d*_0_ = 1, the template of every day is entirely vacant, and only a few requests arrive, which are scheduled for day *d* = 2. We exclude nurse assignment from the simulation, because it is an offline model that has no impact on the KPIs of online appointment scheduling, and it can be evaluated separately. Moreover, the time it takes to solve its MIP is prohibitively long for simulation.

### Fixed-template scheduling heuristic

Figure 4 and Eq. (4) indicate that in the BIP of Eq. (5a), we consider days up to the tolerance outside the flexibility window: one tolerance within the window and one tolerance out of the window. The greatest tolerance is 2 days for patients with *δ*_1,*p*_ or *δ*_2,*p*_ equal to 2. Thus, the farthest day away from the planned day is 2 days out-of-window, which means *OOW*_*p*,*d*_ ∈{0,1,2}. With a maximum deadline of seven days, the number of days that new patients wait, ranges from 1 to 7.

Our computations are in a 64-bit environment. Hence, for signed integers, we have a maximum representable value of only 2^63^ − 1 ≈ 9 × 10^18^ before numerical overflow. We use *L* = 2^42^ as an extremely large value. With *B*_1_ = 23, *B*_2_ = 36, *B*_3_ = 34, *B*_4_ = 32, *B*_5_ = 6, and *B*_6_ = 20, the values of the penalties are as follows:

$$\begin{array}{@{}rcl@{}} &&z{-appointment} \ggg {out-of-window} \ggg {priority} \ggg {wait} \\&& \ggg {overtime} \ggg {idle time} \ggg {start time} \\ &&2^{40} > 2^{38}, 2^{37} \ > \ 2^{34}, 2^{32} \ > \ 2^{30}, \cdots, 2^{24} \ > \ 2^{21}+2^{20}, \\&&\cdots, 0 + 2^{20} \ > \ \Big \lceil2^{19.5} \Big \rceil, \cdots, \Big \lceil 2^{6.0} \Big \rceil\ > \ 34, \cdots, 1 \end{array}$$

In the BIP, the number of *x*, *y*, and *z* variables are $|\mathcal {R}|\times (d_{H}-d_{0})\times S$, $|\mathcal {R}|\times (d_{H}-d_{0})\times S$, and $|\mathcal {R}|\times (d_{H}-d_{0})$, respectively. With the settings of our numerical illustration, $|\mathcal {R}|$ is not expected to be greater than 160 (all 100 slots filled, a *z*-appointment at each of the 33 stations and 27 requests from new patients on a day *d*_0_). *d*_*H*_ − *d*_0_ is at most 46: with a 42-day cycle, the single-day pattern {1}, and *δ*_2,*p*_ = 2, we have at most 42 + 2 × 2 = 46 days after the current day. Thus, the total number of variables is not more than 2 × 160 × 46 × 100 + 160 × 46 = 1,479,360. Let us assume that all of them have the maximum coefficient, *L* = 2^42^, in the objective function. Then the objective value would be less than 6.51 × 10^18^ < 2^62.5^ < 2^63^ − 1. Hence, numerical overflow will not incur.

As there are two types of resources (nurses and stations), idle time is not clearly defined. However, when using a fixed template, idle time can be objectively defined and measured as the number of station-timeslot blocks that are not filled in the original template slots. Thus, we calculate resource utilization as the percentage of the 1091 station-timeslot blocks of the template filled in the *finalized* schedule. However, the reported values for idle time incorporate cancelations while the utilization is measured at the beginning of the day, before any cancelation.

**Fig. 13:**
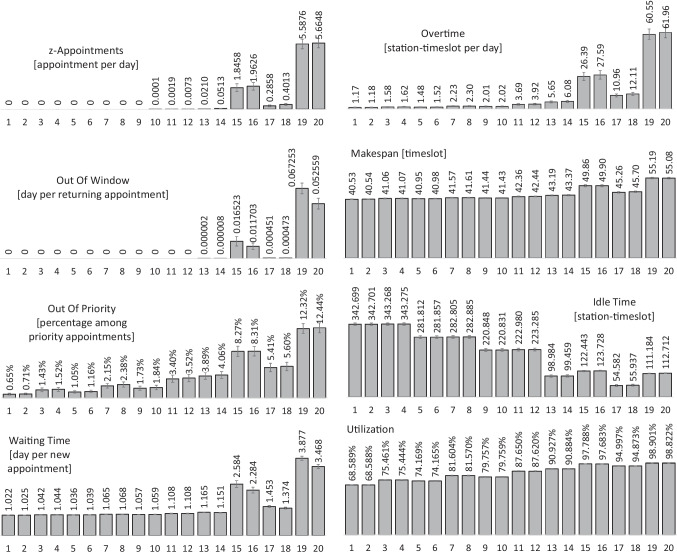
KPI comparison for the 20 cases defined in Table 2

The bottom line for the clinic is to have little overtime with the available resources while treating new patients with few waiting days and returning patients with few OOW days. The mean values and confidence intervals of the KPIs in the steady-state period for the 20 different cases defined in Table 2 are illustrated in Fig. 13.

**Table 2:** The simulated cases (combinations of uncertainty and mode of scheduling) for KPI comparison

Case	*λ*	$\mathbb {P}(cancel)$	Mode
1	6.0	0.00	daily
2	6.0	0.00	immediately
3	6.0	0.10	daily
4	6.0	0.10	immediately
5	6.5	0.00	daily
6	6.5	0.00	immediately
7	6.5	0.10	daily
8	6.5	0.10	immediately
9	7.0	0.00	daily
10	7.0	0.00	immediately
11	7.0	0.10	daily
12	7.0	0.10	immediately
13	8.0	0.00	daily
14	8.0	0.00	immediately
15	8.0	0.10	daily
16	8.0	0.10	immediately
17	8.4	0.00	daily
18	8.4	0.00	immediately
19	8.4	0.10	daily
20	8.4	0.10	immediately

When an appointment is canceled because of the test results, we do not consider using its vacant slot for another appointment. The reason is that even when the blood test is done one day before the appointment and the cancelation is known then, it is not always possible to find another patient whose appointment window and duration fits the canceled slot and is also willing to reschedule. And that is if the other patient can get infusion authorization before the appointment. Hence, cancelation effectively reduces the capacity of the template, and it increases the number of *z*-appointments, the amount of overtime, and the makespan. In Fig. 13, including cancelation in the model (Table 2) worsens the overall performance.

Because of more a priori information at the decision moments in the daily mode, there are fewer *z*-appointments on average per day. Frequent occurrences of *z*-appointments at high Poisson rates indicates that the clinic’s capacity does not match the arrival rate of requests, and it must be increased in the next tactical plan. The impact of levels assigned to penalties in the BIP can be seen across the KPIs. For example, as long as there are not many *z*-appointments, i.e., cases 1 through 14, daily scheduling (odd cases) in general outperforms immediately scheduling (even cases). The reason that in some cases, the daily mode performs slightly worse in some KPIs is a trade-off to improve the more important KPIs, e.g., slightly more waiting time to have less OOW and OOP in cases 13 and 14.

The number of days out-of-window is defined only for returning appointments and measured for the ones that were not canceled. We only allow at most two days OOW in the BIP coefficients before it resorts to a *z*-appointment, which then can only be booked in the zero-cost window. In the extreme case, the number of OOW days per appointment, which is the next most important indicator of performance after *z*-appointments, is 0.067253, where there are cancelations in an overloaded situation. This is roughly 1 day OOW per 15 appointments. Also, as long as the arrival rate is not close to *λ*_*high*_, out-of-priority placement is fairly low: below 10*%* of the priority requests.

Overtime is measured in station-timeslot per day. An overtime of 2.9 is equivalent to on average, only one station running 2.9 timeslots overtime on each day. While in the BIP, we attempt to reduce the amount of overtime, in Fig. 13, we also report the average makespan over the steady-state period to put the values of overtime into perspective. When the arrival rate is close to *λ*_*high*_ and there are cancelations, the makespan exceeds 2.5 hours (ten timeslots) after the regular closing time (*T* = 40).

The idle time reported in Fig. 13 includes idle time due to cancelation as well. It increases when cancelation is incorporated into the model. But at low arrival rates where the schedule already has a lot of idle time, cancelation has almost no impact on idle time. We should note that cancelation does not mean an absolute elimination of the job. The appointment will be postponed for seven days and will be added to the schedule of that day, hence, the apparently mild impact of cancelations, in general.

An important observation is in cases 3, 4, 7, and 8 where demand is 68% or 74% of the template capacity, (e.g., *λ*/*λ*_*high*_ = 6.50/8.77 ≈ 0.74). In those cases, the performance is desirable in both immediately and daily scheduling despite cancelations, which reduce the effective capacity of the template:

- There are no *z*-appointments.
- There are no out-of-window appointments.
- There are on average less than 2.4% out-of-priority placements.
- Average waiting time is less than 1.1 day.
- Overtime is on average less than 2.3 station-timeslots per day while the makespan is on average only 1.6 timeslot after the regular closing time.
- Utilization is on average 75% or 81%.

Although our simulation is for a stylized clinic, it shows that the template and BIP can perform well when the capacity of the template conforms with demand. In practice, for example, chair and nurse utilization can be as low as 52% and 60%, respectively [14]. And total overtime can be as high as 39 hours over 20 days [15], which is equivalent to 7.8 timeslots per day in our model.

### Task scheduling

**Fig. 14:**
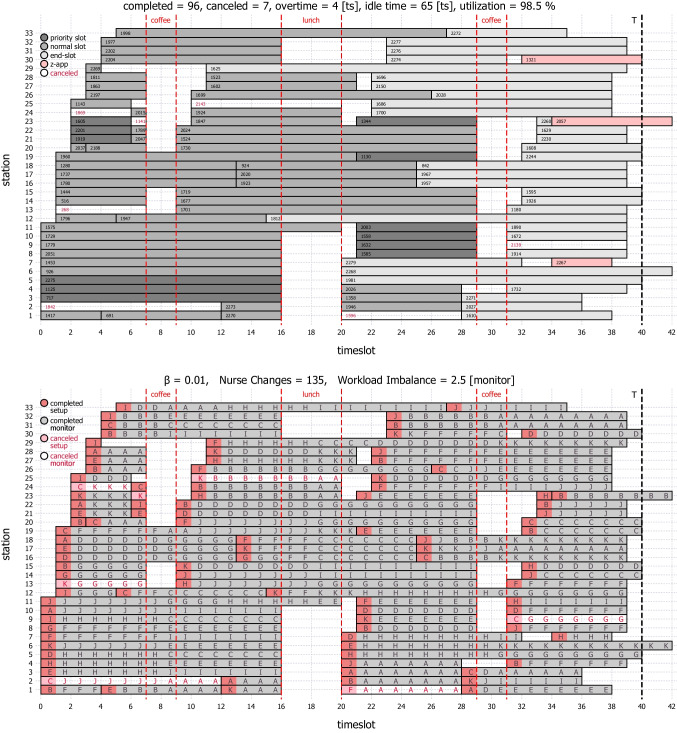
Patients’ appointment schedule and nurses’ task schedule of a day in the steady-state period in hindsight, i.e., after all cancelations are known. The vertical lines demarcate break periods where half of the nurses are not available. The number at the beginning of each slot in the appointment schedule is the patient’s ID, and the letter in each station-timeslot in the task schedule is the nurse’s ID

We have applied the nurse task assignment MIP of Section 4 to the finalized schedule of a day in the steady-state period. Figure 14 shows the appointment and task schedules. No *z*-appointment can be booked to start at *t* = 32, because there are already 6 setups and 19 monitors booked to run then, which require 6 + ⌈19/4⌉ = 11 nurses. Hence, no nurse is available to set up a *z*-appointment, and the next availability is at *t* = 33. There are 1081 station-timeslot blocks booked for 103 patients to be taken care of by the 11 nurses. Thus, Γ = (3 × 103 + 1081)/11 = 126.4.

With *β* = 0, the minimum of the workload part of the objective function is 2.5. With *β* = 1, the minimum of the nurse-change part of the objective function is 226 (with 134 nurse changes). The task schedule in Fig. 14 is generated with *β* = 0.01. There are 135 nurse changes during appointments, which translates to on average one nurse change after (1081 × 15)/135 ≈ 120 minutes of continuous care by the same nurse. Sixty of the nurse changes are unavoidable because of the coffee and lunch breaks. The 7-hour appointment of patient-1625 at station-29 has only four nurse changes, where one of them is for the lunch of nurse-H and another is for the coffee of nurse-D. Each of the 11 nurses (*A*,⋯ , *K*) would have 9.1*%* of the total workload of the day, had there been no cancelations. In the task schedule, the workload is 127 monitors for four nurses and 126 monitors for the remaining seven with a total workload imbalance of 2.5 monitors.

The MIP gap is 4.9% after 3906 seconds of solve-time. The appointment schedule in Fig. 14 with *P* = 103 patients is representative of a clinic in a large metropolitan area. For more common clinic sizes, e.g., with 40 to 60 patients on average per day, the nurse task scheduling MIP model solves notably faster.

## Concluding remarks

We have demonstrated the effectiveness of a fixed template for incorporating the interests of various stakeholders in online scheduling. Our proposed heuristic is formulated as a binary integer program to book appointments as requests arrive. The heuristic is based on scheduling principles that are in alignment with the interests of stakeholders. The following are examples of such principles for drug administration appointments in outpatient chemotherapy:

- little indirect waiting time for new patients
- little side effects and diminishing results for returning patients
- few non-allocated placements for priority patients
- little overtime for nurses and little idle time for the clinic

The scheduling principles are embedded in the cost coefficients of the BIP objective function, and they can be adjusted based on the preferences of the clinic or the patients. For example, to incorporate a patient’s time preference, we can reduce the cost coefficients of the slots that start in the morning or in the afternoon, or give extremely high cost coefficients to variables with specific day and slot indexes. Another example is to assign specialized nurses to specific patients, e.g., clinical trial patients, by giving extremely high cost coefficients to variables with indexes of other nurses.

The same BIP can be used for both immediately and daily responding to requests. However, because of having more a priori information, daily scheduling has more flexibility for incorporating managerial preferences. For example, all returning patients can be scheduled first, in order to have their appointments in the ZCWs as many as possible. Another example is to give urgency to a select few new patients who have to start their treatment on short notice, e.g., the day after the request.

Our simulations show that when demand is around 70% of the template capacity, i.e., *λ*/*λ*_*high*_ ≈ 0.70, the performance is acceptable in both immediately and daily scheduling across various criteria for multiple stakeholders, also when there are cancelations, which reduce the effective capacity of the template.

We have also illustrated a bicriterion mixed integer program for assigning tasks offline after the online appointment schedule is closed in the case of chemotherapy drug administration. The goal is to have equal workload among nurses with very few during-appointment nurse changes, which makes the service less confusing and safer.

The template needs to be updated at a tactical planning level. The amount of overtime and the number of added slots (*z*-appointments) or the amount of idle time and shorter than the closing time makespan in the current tactical plan can be used to increase or decrease the capacity of the template for the next tactical period. However, since it is safer to design the template matching the histogram of demand with the most number of slots for the available resources, idle time may not be a strong indicator for decreasing the capacity. That is why we give it a low level of penalty in the BIP. For chemotherapy with treatments lasting more than half a year, updating the template once a year can be enough. For services with seasonality or non-recurrent services, it may be updated every one to three months.

The heuristic can be used for other services where there is an initial setup followed by simultaneous monitoring, e.g., computer-based standardized test centers offering tests with various durations. In addition, our method of incorporating online scheduling principles as soft constraints into the objective function of a mathematical program for filling a fixed template can be used in other application areas. Part of the scheduling requirements are embedded in the template, e.g., the starting times of slots in the template are limited by the setup and monitoring hard constraints, or the template is designed for minimum makespan for the given slots. In applications where no setup or resource sharing is required, e.g., primary care or specialist services in healthcare other than chemotherapy, the template is designed without those requirements but the same or other scheduling principles can be incorporated into the BIP for filling the template online. The deterministic model (deterministic durations, no cancelations, and punctual arrivals) can be adopted for less uncertain applications, e.g., cloud services or manufacturing with reliable machinery.

### Limitations, countermeasures, and future research

We have assumed a Poisson arrival process for patients being referred to the clinic for their first appointments. The simulations have shown that the performance of the heuristic depends on the ratio between the new-patient arrival rate and the new-patient service rate of the template (*λ*/*λ*_*high*_). Though the template is designed based on the relative frequencies of past demand (Fig. 11), the empirical arrival process is not incorporated into the model. Hence, recording empirical data as to the new patient arrival rate *λ*, number of planned days per cycle $\mathbb {E}(D)$, number of cycles $\mathbb {E}(C)$, and $\mathbb {P}(cancel)$ is needed for effective and efficient resource allocation. However, when such data are not available, these parameters can be estimated using expert opinion (from nurses and oncologists) and the total number of appointments in the past couple of years. Since the cost of a station can be notably lower than the cost of a nurse FTE, allocating close to (*N* − 1)*M* + 1 stations [12] for the number of nurses permanently available during the tactical plan lowers the risk of undercapacity until empirical data are collected for the next tactical plan.

We have assumed deterministic appointment durations (job processing times). If there are uncertainties in the durations, the template should be designed based on the histogram of actual durations in the past rather than booked appointments or procedural durations, e.g., instead of those indicated in treatment protocols. However, a slight amount of buffer time that depends on the appointment and applicable risk factors can be added to the end of appointments before forming the histogram. We have also assumed that patients are punctual, which can be justified at many chemotherapy clinics by patient support programs. Thus, our heuristic is a proactive operational model [11, 16]. We note that the above mentioned buffer time at the end of slots cannot mitigate unpunctual arrivals in chemotherapy because of the setup constraint on the nurses. Hence, reactive operational countermeasures such as rescheduling policies for delays can be devised in follow-up research. In addition, procedures can be updated based on historical durations and the likelihood of adverse circumstances (e.g., allergic reactions) that may prolong the appointment durations. Appointments with more likelihood of adverse circumstances can be placed in end-slots to avoid the risk of delaying the starting times of other appointments.

We have assumed that the service is available every day including weekends and holidays. In chemotherapy, this has no impact on new patients. It simply implies that the deadline is defined over the working days. For returning patients, however, an appointment may fall out of the ZCW because of skipping one or more days. In those cases, OOW appointments may be avoided by adjusting the desired day with *δ*_1,*p*_ = *δ*_2,*p*_ = 0 for that specific appointment.

Since short notice cancelation can increase the workload imbalance on the day, the cumulative workload of the resources over one or more weeks can be used to balance the total workload over a period. For example, the workload threshold of overworked resources can be lowered or their workload can be limited to a certain amount in the next task schedules until the total workloads reach an acceptable balance among themselves.

## Footnotes

1 *Indirect waiting time* is between the moment the patient asks for an appointment and the start of the appointment, in contrast to *direct waiting time*, which is the duration of the time that the patient was required to be in the health center but received no service, i.e., appointments starting with delays on the day.
